# CFIHL: a variety of chlorophyll a fluorescence transient image datasets of hydroponic lettuce

**DOI:** 10.3389/fpls.2024.1414324

**Published:** 2024-09-12

**Authors:** Yiyu Jiang, Yu Tan, Fang Ji, Daobilige Su, Shuo Wang, Lina Zhang, Qing Zhou

**Affiliations:** ^1^ College of Engineering, China Agricultural University, Beijing, China; ^2^ College of Water Resources and Civil Engineering, China Agricultural University, Beijing, China

**Keywords:** chlorophyll a fluorescence, hydroponic lettuce, parameterized image, image fusion, semantic segmentation

## 1 Introduction

Artificial light plant factories ensure year-round stable vegetable production, and vertical multi-cultivation racks can boost vegetable yields (Toyoki et al., 2020). Seedling diagnosis technology is crucial for early identification and removal of weak seedlings, aiming to cut costs in plant factories. This technology relies on seedling biological data to predict growth and effectively manage underperforming seedlings.

Chlorophyll a fluorescence (ChlF) is a long-wavelength signal emitted by plants following light absorption during photosynthesis, directly associated with photosynthetic efficiency (Huipeng, 2023). ChlF is transient and diminishes over time after excitation. Cameras can capture different parameterized images of ChlF depending on excitation wavelength, intensity, and mode (Yao et al., 2018). Parameters like photosynthetic quantum yield (Φ_PSII_), photochemical quenching (qP), and Photosystem II electron transport rate (ETR(II)) are vital for assessing plant productivity. Higher qP, Φ_PSII_, and ETR(II) in lettuce indicate greater light utilization efficiency and higher plant yield. Therefore, ChlF imaging is widely used for monitoring photosynthetic performance in plants (Thoren et al., 2010). ChlF imaging technology can assess heterogeneous distribution of photosynthetic activity Lichtenthaler et al. (2013), Xia et al. (2018). This includes early detection of herbicide application, nutrient deficiency, drought stress, photorespiratory mutants, improved photosynthesis, freezing tolerance, insect herbivory, leaf fungal infection, and ozone damage (Lawson and Vialet-Chabrand, 2018). Therefore, it is necessary to collect ChlF images to realize the study of plant growth stress and plant yield estimation.

At present, there are few plant chlorophyll fluorescence datasets, with most focusing on stress identification in Arabidopsis crops. To enable the chlorophyll fluorescence system to track photosynthesis in a single leaf, Ruiz et al. (2024) proposed a chlorophyll fluorescence dataset for Arabidopsis plants at different developmental stages and used an improved Mask R-CNN network to segment Arabidopsis leaves, accurately tracking changes in leaf photosynthesis. The Computer Vision Problems in Plant Phenotyping (CVPPP) dataset consists of 783 RGB images of wild Arabidopsis and tobacco plants, used for testing algorithms such as plant segmentation, leaf segmentation, and leaf counting (Minervini et al., 2016). Additionally, Rousseau et al. (2013) collected 49 fluorescence images of Arabidopsis inoculated with sterile water, naming it the ‘Real-Fluo-Healthy’ dataset. However, this dataset only contains images of healthy Arabidopsis and cannot provide a basis for disease diagnosis. Therefore, Rousseau et al. further used Arabidopsis plants inoculated with the DC3000 bacterial strain as the research object, performing chlorophyll fluorescence imaging on each culture dish every two days to obtain TIFF images of Fo, Fm, and Fv/Fm for judging the status of diseased Arabidopsis. In addition, Pavicic et al. (2021) proposed an annotated dataset of diseased Arabidopsis fluorescence images and a threshold segmentation algorithm to segment the normal and diseased leaf areas in the images for disease diagnosis. Currently, there is a lack of similar chlorophyll fluorescence datasets for hydroponic lettuce research, which mainly uses RGB image data to evaluate lettuce growth status. Islam et al. (2024) used an improved Mask R-CNN method to detect and segment lettuce seedlings from the background of the seedling growth tray and estimate seedling growth. Abidi et al. (2024) collected an RGB image dataset of nutrient deficiencies in hydroponic lettuce and proposed a deep learning image processing method to classify these deficiencies. However, RGB images can only capture superficial color and texture information, making it difficult to obtain internal physiological parameters of lettuce leaves and accurately judge lettuce growth status. Therefore, this paper collects a ChlF dataset of hydroponic plants and tests region of interest extraction algorithms for fluorescence images, providing data for accurately estimating lettuce seedling status using ChlF technology.

Hydroponically grown lettuce often uses sponges for anchorage, making it more challenging to extract the fluorescent area compared to plants grown in substrates, due to the common presence of microbes or green algae within the cultivation sponges (Weizhong, 2023). During the capture of ChlF systems, the fluorescence from microbes or green algae present in the cultivation sponges is also captured, resulting in minimal differences between them in grayscale images taken under blue light, affecting the extraction of areas of interest in the ChlF images of hydroponic lettuce seedlings. In microbial fluorescence images, such as that of Chlamydomonas reinhardtii, exposure to excitation light produces a lower level of Non-Photochemical Quenching (NPQ). In the chloroplasts of algae like Chlorella or Chlamydomonas, effective carbohydrate metabolism decomposition leads to a much reduced redox balance compared to plant chloroplasts (Alric, 2010). Differences exist in the fluorescence parameters under ChlF induction kinetics between lettuce and green algae (Papageorgiou et al., 2007). However, the threshold segmentation algorithm cannot determine the category of the area. In practice, the grayscale of the sponge block of green algae in the initial fluorescence image is very similar to that of the seedling canopy, leading to mis-segmentation of areas with similar grayscales. Given the excellent feature extraction and generalization performance of deep learning algorithms in image recognition, a deep learning semantic segmentation algorithm is considered for extracting the seedling canopy area from a single fluorescence image. Testing has shown that fusing multiple fluorescence images can improve the extraction accuracy of the seedling canopy area, effectively reduce background noise interference, and more accurately extract the fluorescence values. Image fusion, by integrating information from multiple sources with complementary datasets to enhance a single image (Qu et al., 2024), makes the generated images more detailed and reliable through the combination of key information from multiple source images (Karim et al., 2023). At this stage, it has been proposed to introduce accuracy of advanced visual tasks such as detection, recognition, and segmentation into the design of the loss function during the fusion phase (Liu et al., 2023; Ma et al., 2023), guiding the fusion process from a decision-making level to achieve better segmentation results (James and Dasarathy, 2014).

This article introduces the Hydroponic Lettuce Seedling ChlF Dataset. The dataset comprises various transient images of ChlF from different cultivation states, with annotations marking the positions of the entire lettuce canopy projection. The aim is to accelerate the development of algorithms for extracting ChlF areas in hydroponically grown lettuce seedlings. The data for this article is publicly released at https://github.com/Yiyu-Jiang/CFIHL. For user convenience, the original.igr data files (which can be exported as chlorophyll images, tables, documents) are provided, along with the corresponding annotation JSON files.

The primary contributions of this dataset are summarized as follows:

(1) The CF-imager has provided a ChlF image set of the whole hydroponic lettuce seedlings, with the dataset being approximately 1.3 G in size. The dataset includes more than 300 sets of images, each set containing ten transient ChlF images. The dataset offers a variety of physiological images of plants and annotations for individual seedlings.
(2) Compared to the existing lettuce seedling datasets, this paper has conducted tests with multiple image algorithms, comparing threshold segmentation and deep learning algorithms, as well as the segmentation effects of images before and after fusion. The fused image can extract the ChlF value of lettuce more accurately, and can further accurately obtain parameters such as photochemical quantum yield, thereby reflecting the photosynthetic utilization efficiency and lettuce yield.

## 2 Materials and methods

### 2.1 Data acquisition

Lettuce was cultivated in the artificial light plant factory of China Agricultural University in 2022 and 2023. During the cultivation of hydroponic lettuce, we used an LED lamp (WR-16W) with an R ratio of 1.2, a light intensity of 200 *µ*molm^−2^s^−1^, and a photoperiod of 16 hd^−1^. The temperature during the light period was 22 ± 1°C, with a relative humidity of 70 ± 5% and a CO_2_ concentration of 800 ± 50*µ*molmol^−1^. During the dark period, the temperature was 18 ± 1°C, the relative humidity was 65 ± 10%, and the CO_2_ concentration was not controlled. The seedling raising period for hydroponic lettuce was 20 days. The nutrient solution used was the Yamazaki lettuce formula Yan (2019), with an EC of 1.0-1.2 mScm^−1^ and a pH adjusted to 6.0-6.5 mol*/*L.

**Figure 1:**
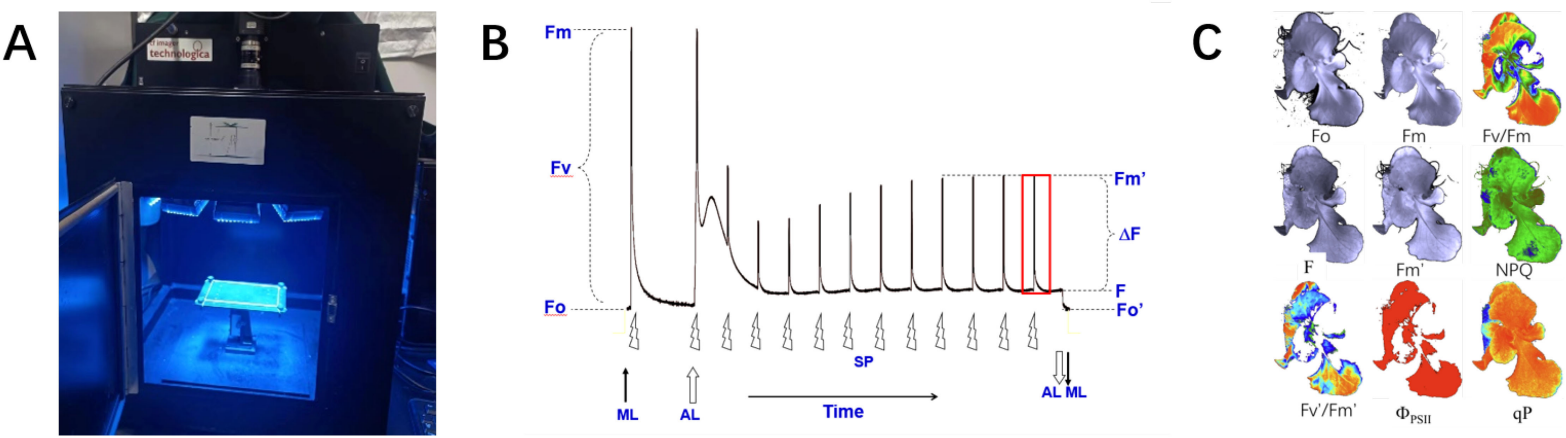
Details of data acquisition. **(A)** ChlF imaging equipment. **(B)** Schematic diagram of ChlF measurement principle. **(C)** ChlF result images.

In this paper, the seedlings grown for about 21 days were placed in the CF-Imager(Technologica, UK) instrument, then photographed, as shown in **Figure 1A**. The CF-Imager system uses a high-performance AVT Stingray SXGA+ low-noise line scan CCD camera with a resolution of 1392 × 1040. When collecting ChlF images, the camera needs to respond quickly, which sacrifices some resolution. Therefore, the image resolution was set to 696 × 519. The imaging frequency is 50 FPS, and the readout noise is less than 12e. This ChlF imaging system used 470 nm blue light as the excitation light. Based on the principles of slow fluorescence kinetics, the detection light was set to 5 *µ*molm^−2^s^−1^, the actinic light to 200 *µ*molm^−2^s^−1^, and the saturating pulse light to 6000 *µ*molm^−2^s^−1^ with a duration of 800 ms, establishing the excitation process (Daquan, 2002).

The measurement principle is illustrated in **Figure 1B**. After 30 minutes in the dark, a fully dark-adapted leaf was exposed to measuring light. Within 1-2 minutes, as the fluorescence level stabilized, the minimum initial fluorescence Fo image was obtained. Then, a saturating pulse light was applied and turned off after one pulse to obtain the maximum fluorescence image Fm. Therefore, the fluorescence parameter image Fv/Fm (Fv=Fm-Fo) can be calculated, representing the potential photochemical efficiency of PSII.

Secondly, the actinic light of 200 *µ*molm^−2^s^−1^ was turned on to induce leaf photosynthesis. After a few minutes, the leaf’s photosynthetic rate reached a steady state, and the steady-state fluorescence(Fs) image was obtained. At this point, a pulse of saturating light was applied and then turned off to obtain the maximum fluorescence image under light Fm’. The actual quantum efficiency of PSII(Φ_PSII_) and the non-photochemical quenching coefficient (NPQ), defined as (Fm -Fm’)/Fm’, were then calculated.

Using the method described above to stimulate fluorescence in vegetables, various ChlF transient images were obtained, as shown in **Figure 1C**, all with a resolution of 696 × 519. The ChlF images are essentially grayscale images with a single channel. For intuitive description, this paper maps the grayscale values of some ChlF images to the RGB color space (Oxborough, 2004), rendering them as pseudocolor images.

The main ChlF parameters and descriptions are shown in **Table 1**:

**Table 1:** Commonly abbreviated and described ChlF parameters.

Parameter	Formula	Description
PSII	\	Photosystem II: it is a photosynthetic unit in the thylakoid membrane that contains two light-harvesting complexes and a light reaction center.
Fo	\	Minimum initial fluorescence
Fm	\	Maximum fluorescence
Fm’	\	Maximum fluorescence under light
Fs	\	Steady state fluorescence
NPQ	Fm/Fm’-1	Non-photochemical quenching: estimates the rate constant for heat loss from PSII.
Fv/Fm	(Fm-Fo)/Fm	Maximum quantum efficiency of PSII photochemistry.
Fv’/Fm’	(Fm’-Fo’)/Fm’	Maximum efficiency of PSII photochemistry in the light, if all centers were open.
Φ_PSII_	(Fm’-F’)/Fm’	PSII operating efficiency: the quantum efficiency of PSII electron transport in the light.
qP	(Fm’-Fs)/Fm’-Fo’	Photochemical quenching: relates PSII maximum efficiency to operating efficiency. Non-linearly related to proportion of PSII centers that are open. See qL.

This list is only used to identify the most common parameters, as detailed in the review (Murchie and Lawson, 2013).

### 2.2 Data structure and annotation

**Figure 2:**
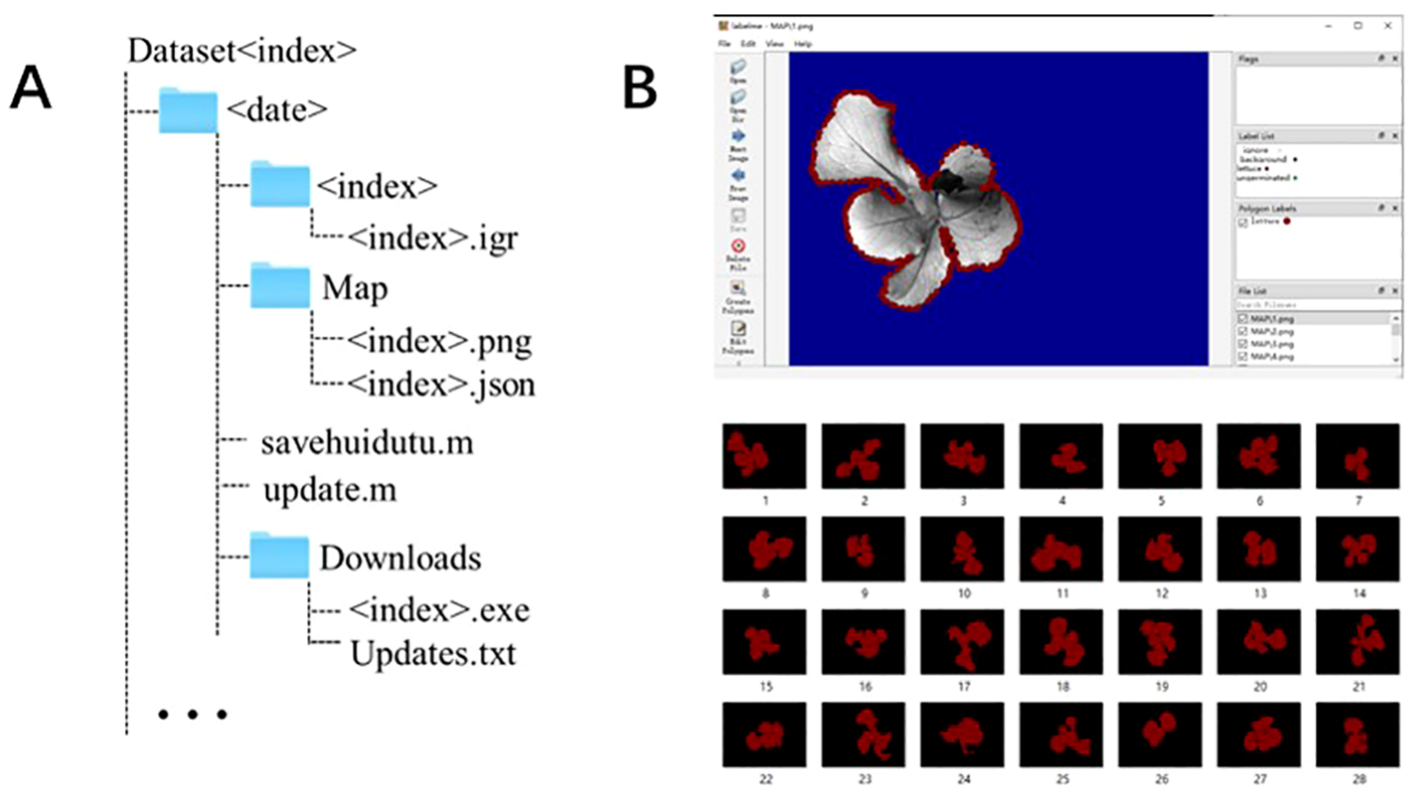
Dataset format and image annotation. **(A)** Folder structure for dataset. The term <date> refers to the time of the acquisition of a dataset, while the term <index> identifies each piece of data. **(B)** Example of image annotation.

The CF-Imager can capture 10 types of images: the initial grayscale image (Igi), Fo, Fm, Fv/Fm, Fm’, Fs, NPQ, Fv’/Fm’,Φ_PSII_ and qP. Detailed information about the dataset is shown in **Figure 2A**. To help users differentiate the fluorescence images of seedlings in various states, the raw fluorescence data are stored in separate folders in chronological order, totaling approximately 270 sets of image data. Additionally, to enrich the dataset and investigate the changes in ChlF parameters under different growth conditions of lettuce. This dataset collected images under three distinct environments: nutrient deficiency (only ultrapure water, no nutrients), extremely low light intensity (only 10 - 20 *µ*molm^−2^s^−1^ ambient light), and abnormal EC and pH (EC is about 900 mS/cm, pH is about 7.5 mol/L).

Image segmentation aims to accurately extract the ChlF parameters of the seedling canopy. Image annotation is a prerequisite for training deep learning-based image segmentation models. It provides the training model with prior knowledge of the seedling canopy area within the image. This prior knowledge helps establish the main parameters of the model, enabling the recognition of similar images. This study uses LabelImg (https://github.com/tzutalin/labelimg) to annotate the seedling canopy area in the fluorescence images of the dataset. The seedling canopy area is labeled as ‘lettuce’, generating a label mask image and a JSON file for further neural network model training, as shown in **Figure 2B**.

### 2.3 Image fusion algorithm and semantic segmentation algorithm test

#### 2.3.1 Algorithm introduction

This paper tests extraction algorithms for various lettuce canopy projection areas using the constructed ChlF transient image dataset. It eliminates fluorescence interference caused by microorganisms or green algae in the background sponge and explores effective methods to accurately obtain ChlF parameters of the seedling canopy. This provides a basis for further evaluation of lettuce growth status.

First, a comparison between threshold segmentation and deep learning image segmentation algorithms was conducted on a single ChlF image. Early research on image segmentation mainly focused on threshold segmentation methods, where one or several threshold values divide the grayscale histogram into several classes. It was believed that the grayscale value of the seedling canopy was consistent and distinct from the background area. The existing fluorescence systems mainly use threshold segmentation (Lawson and Vialet-Chabrand, 2018). However, threshold segmentation algorithms cannot accurately categorize different areas. In practice, the grayscale of green algae on the sponge is very similar to that of the seedling canopy in the initial fluorescence grayscale image, leading to mis-segmentation. Therefore, this paper introduces a more adaptable deep learning segmentation algorithm. Deep learning has been widely used in image recognition due to its excellent feature extraction and generalization capabilities, achieving good segmentation results (Li et al., 2021). Among them, the DeepLab series is one of the most popular image segmentation models (Minaee et al., 2022). Therefore, this paper selects the DeepLabv3+ network (Chen et al., 2018), which has the best segmentation performance in the DeepLab series, as the deep learning semantic segmentation test algorithm.

Then, the traditional fusion algorithm and the SeAFusion algorithm based on advanced visual tasks were tested on multiple images, and the deep learning segmentation algorithm was used to segment and compare the images before and after fusion. In addition to single image segmentation, this paper also tested multiple image fusion methods. Image fusion technology can be divided into several types, namely remote sensing image fusion, multi-exposure image fusion, and visible light and infrared image fusion. Among these, visible light and infrared image fusion is one of the most commonly used types. The main purpose of visible light and infrared image fusion is to extract the detail information from visible light and the contrast information from infrared image, which is similar to the goal of fluorescence image fusion. Therefore, this paper selected four recently released image fusion algorithms CNN, IFEVIP, LatLRR, MST-SR (Zhang et al., 2020).

However, these fusion algorithms primarily focus on the visual quality and statistical indicators of the fused image, while ignoring the needs of advanced visual tasks (i.e. classification, localization, detection and segmentation). Therefore, this paper tested the image fusion algorithm named SeAFusion (Tang et al., 2022) combined with segmentation tasks. The main concept of the SeAFusion algorithm is to generate a fused image by passing the source image through a fusion network. Then inputting the fused image into a segmentation network to obtain the segmentation result, where the segmentation network used in the SeAFusion algorithm is DeepLabv3+.

#### 2.3.2 Image segmentation evaluation indicators

This study uses Precision, Recall and intersection over union (IoU) as evaluation indicators for training target detection models. The range of these three evaluation metrics is [0, 1], the detailed descriptions are provided in **Table 2**. In **Table 2**, ‘TP’ represents true positives, indicating the number of samples correctly classified as positive. ‘FP’ represents false positives, indicating the number of samples incorrectly classified as positive. ‘FN’ represents false negatives, indicating the number of samples incorrectly classified as negative.

**Table 2:** Image segmentation algorithm evaluation indicators.

Parameter	Formula	Describe
Precision	$\frac{\text{TP}}{\text{TP}+\text{FP}}\times 100\%$	Precision indicates the percentage of correctly identified pixels among the total number of identified pixels.
Recall	$\frac{\text{TP}}{\text{TP}+\text{FN}}\times 100\%$	Recall indicates the percentage of correctly identified pixels that meet the requirements to the total number of pixels in the test set.
IoU	$\frac{\text{TP}-\text{FP}}{\text{TP}+\text{FN}}\times 100\%$	IoU (Intersection over Union) is the ratio of the number of elements in the intersection of two sets to the number of elements in their union.

#### 2.3.3 Implementation details

All models in this study were trained on a single GPU. The hardware configuration for training and testing the network models includes an i9-12900K CPU, NVIDIA GeForce RTX 3070 GPU, and 32GB DDR4 RAM. The dataset was randomly divided into training, test, and validation sets in an 8:1:1 ratio. The input size of each fluorescence image is 696 × 519 pixels. The DeepLabv3+ algorithm (Yu et al., 2024) sets the number of iterations (Epochs) to 300 times, with an initial learning rate of 0.001 and a batch size of 4. SGD is used as the optimizer, with a weight decay of 0.0001 and a momentum of 0.9. The training weights are verified and saved every 5 iterations.

## 3 Result

**Figure 3:**
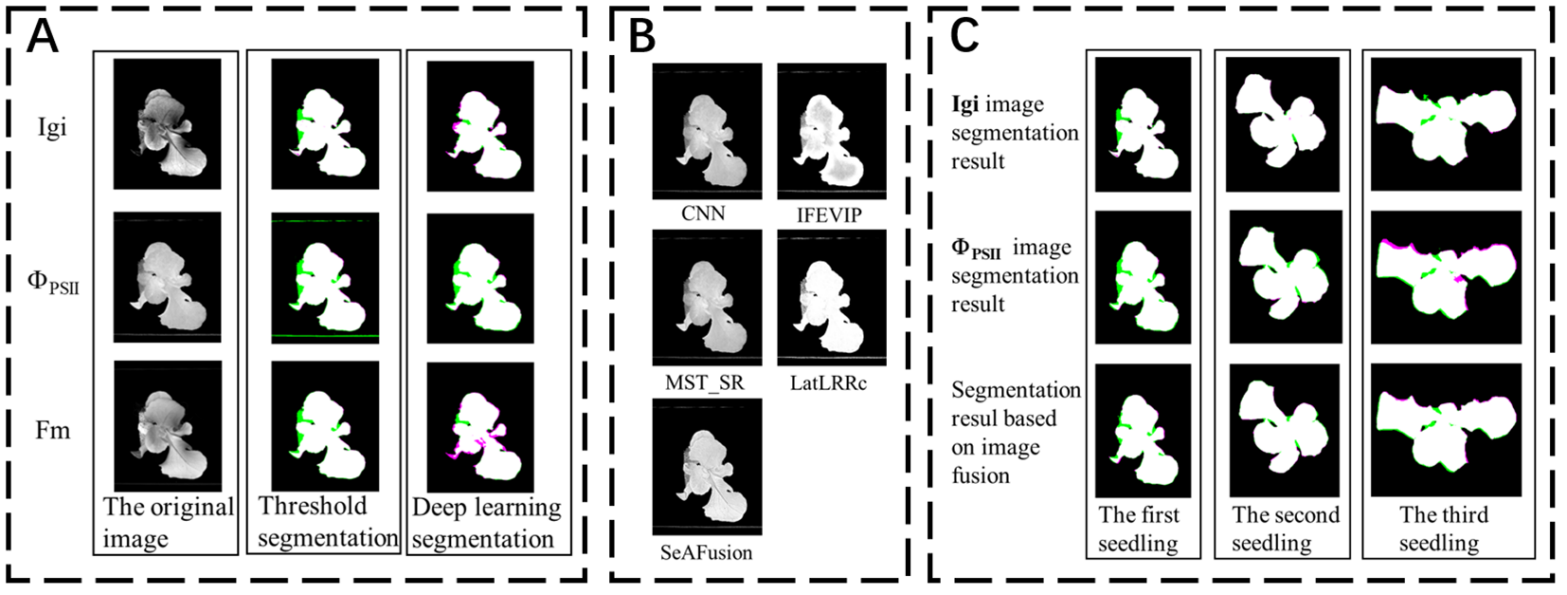
**(A)** Comparison of segmentation results of different fluorescence images using different segmentation algorithms. **(B)** Results of fusion algorithms using 5 other infrared and visible light fusion algorithms. **(C)** Deep learning image segmentation results before and after fusion.

In this paper, the automatic threshold segmentation method (Zhao et al., 2023) and the deep learning DeepLabv3+ network are used for the initial grayscale image (Igi), photochemical quantum yield (Φ_PSII_) and maximum fluorescence (Fm) images. The segmentation results are shown in **Figure 3A**. Visualizing the segmentation results allows us to intuitively assess the accuracy of each algorithm in segmenting the edges of lettuce seedlings. The green areas in the figure indicate non-canopy areas mistakenly identified as canopy areas, while the magenta areas indicate canopy areas not recognized by the segmentation algorithm.

The image segmentation results indicator results are shown in **Table 3**. Among them, the threshold segmentation algorithm has the best segmentation effect for the Fm parameterized image with an IoU of 84.89%, but there is still a 15% error with the actual canopy. The deep learning DeepLabv3+ network has higher segmentation accuracy in most images than the threshold segmentation method, and the best segmentation effect is for the Igi image, with an IoU of 87.27%, an increase of about 2%. Automatic threshold segmentation can roughly distinguish the seedling canopy from the background. However, the presence of green algae can cause the background threshold to be similar to that of the seedling, leading to incorrect segmentation. In contrast, deep learning methods automatically extract features and are wellsuited for segmenting complex scenes with similar pixels. Consequently, deep learning has shown improved segmentation effectiveness compared to simple grayscale threshold segmentation.

**Table 3:** Comparison of image segmentation results.

Algorithm		Precision/%	Recall/%	IoU/%
Threshold segmentation	Igi Φ_PSII_	88.74 86.00	99.36 88.95	83.02 77.69
	Fm	86.82	97.45	84.89
Deeplabv3+ algorithm	Igi Φ_PSII_	89.57 83.80	97.13 93.36	87.27 80.93
	Fm	85.81	96.94	83.55

This study uses the DeepLabv3+ algorithm to segment the seedling canopy area of fluorescence images. The Precision, Recall, and IoU are slightly improved compared to threshold segmentation, but the IoU is still below 90%. Therefore, it is necessary to consider other segmentation methods to improve the accuracy of seedling canopy segmentation. Considering the complementarity between fluorescence images, this study first fuses the ChlF transient image set. Then inputs the fused image into the segmentation network for canopy segmentation.

The fusion results of Igi and Φ_PSII_ for the same seedling using four popular fusion algorithms (Zhang et al., 2020) and the SeAFusion algorithm are shown in **Figure 3B**. The segmentation visualization results of the DeepLabv3+ network before and after the fusion of three seedlings are shown in **Figure 3C**. This paper uses indicators such as entropy (EN), standard deviation (SD), spatial frequency (SF) and sum of difference correlations (SCD) to characterize the effect of image fusion (Zhang et al., 2019). The higher EN and SD indicate that the method can better preserve the source image. These images contain a large amount of information and have high pixel contrast (Liu et al., 2023).The comparison results of each fusion algorithm are shown in **Table 4**. Among them, the fusion image texture information obtained by the CNN, IFEVIP, LatLRR, and MST_SR algorithms has a greater loss. The fused image obtained by the SeAFusion algorithm not only ensures the contrast between the seedling canopy and the sponge block, but also fully retains the seedling texture. Combined with the visualization results in **Figure 3B**, the SeAFusion algorithm in **Table 4** has higher AG, SD, and EN parameter values and clearer edges compared to other fusion algorithms from a visual perspective.

**Table 4:** Fusion algorithm comparison.

Indicators Algorithm	AG	SF	SD	SCD	Q^abf^	EN
CNN	1.63	16.37	6.16	NaN^1^	0.98	1.35
IFEVIP	1.76	21.22	6.17	1.70	0.67	1.23
LatLRR	1.93	25.52	6.17	1.72	0.40	0.93
MST-SR	1.63	16.37	6.16	NaN^1^	0.98	1.35
SeAFusion	3.11	0.054	9.23	1.62	0.49	3.76

^1^NaN (Not a Number) is a value in computer science representing an undefined or unrepresentable value.

Finally, to verify that the fused image segmentation effect is better, this paper uses the Deeplabv3+ algorithm to compare the segmentation results of the fused images of the two fusion methods (Igi is fused with Φ_PSII_ and Fm images respectively), and the results are shown in **Table 5**. Compared with the segmentation results in **Table 3**, the fusion Precision, Recall and IoU ratio parameters are increased by 0.25%, 0.35% and 0.56% respectively, which is helpful for the accurate extraction of fluorescent areas of interest such as the lettuce canopy. Among them, the fluorescent image segmentation results of three seedlings are shown in **Figure 3C**. There are many wrongly segmented areas (green and magenta areas) in the seedling canopy segmentation using the Deeplabv3+ algorithm without image fusion, while the wrongly segmented areas (green and magenta areas) are significantly reduced after fusing Igi and Φ_PSII_. This is because the SeAFusion algorithm transmits the semantic information required for high-level visual tasks (segmentation) back to the fusion network, thereby enabling the fusion network to effectively retain the semantic information in the source image.

**Table 5:** Comparison of image segmentation results.

Indicators Image collection	Precision/%	Recall/%	IoU/%
Igi and Φ_PSII_ images are fusioned	95.35	96.17	91.86
Igi and Fm images are fusioned	95.60	96.52	92.42

## 4 Conclusion

This paper presents a ChlF dataset of hydroponic lettuce seedlings, featuring diverse transient images under various cultural conditions. Additionally, the study compares the effectiveness of the threshold segmentation algorithm with the deep learning-based Deeplabv3+ algorithm for extracting the seedling canopy. Owing to its superior feature extraction capability, the Deeplabv3+ algorithm outperforms the threshold segmentation method in various fluorescence parameterized images. However, its IoU score remains below 90%. Consequently, various image fusion algorithms were tested, and the fused images were semantically segmented using the Deeplabv3+ algorithm. The results indicate that the fused images provide better segmentation of the lettuce canopy. The dataset introduced in this paper supports the evaluation of lettuce growth status based on ChlF data and offers methods for accurately extracting fluorescence image parameters of lettuce.

## 5 Discussion

Inspired by the Arabidopsis dataset, this paper proposes a new hydroponic lettuce seedling ChlF dataset to address the lack of similar datasets. Additionally, the paper deeply analyzes the issue of extracting plant canopy areas from ChlF images in the dataset. The ChlF system captures both lettuce and green algae fluorescence simultaneously, with the initial fluorescence image showing similar values for the seedling canopy and the background. Therefore, this paper uses the deep learning Deeplabv3+ algorithm to leverage its powerful feature extraction capabilities to improve the segmentation of the seedling canopy.

However, due to limited information in a single image, the paper also considers the fluorescence value differences between the seedling canopy and the background, adopting canopy segmentation after image fusion. Further testing of multiple image fusion algorithms revealed that the fused images provided better segmentation of the lettuce canopy. Thus, using complementary information from different fluorescence images improves the segmentation of the seedling canopy’s edge contours.

There are some limitations in this study. For example, only some image fusion methods were tested during image fusion, and all fusion combinations were not enumerated due to the limited length of the article. Only two fluorescence parameter images were considered as input, and multiple image inputs were not considered. The fusion algorithm tested in this study is designed for merging infrared and visible light. It is recommended to design a fusion method suitable for the fusion of multiple fluorescence images in the future to help improve the acquisition of ChlF values. In addition, for the current dataset, future research should further expand the sample range and explore applications in different objects and scenarios such as plant mesophyll and vein segmentation, and segmentation of different types of cells in fluorescence images, in order to prove that the fusion of different fluorescence images has a wide range of applications and is worthy of in-depth research.

## Data Availability

The datasets presented in this study can be found in online repositories. The names of the repository/repositories and accession number(s) can be found below: https://github.com/Yiyu-Jiang/CFIHL.
