# Supplementary material for: CFIHL: a variety of chlorophyll a fluorescence transient image datasets of hydroponic lettuce
Source: Front Plant Sci. 2024 Sep 12;15:1414324. doi: 10.3389/fpls.2024.1414324 (PMC11428101; doi:10.3389/fpls.2024.1414324)
Supplement: Supplementary file 1 [file DataSheet1.pdf]

Parameter	Formula	Description
AG	$AG=\frac{1}{(M-1)(N-1)}\times\sum_{i=1}^{(M-1)}\sum_{j=1}^{(N-1)}\sqrt{\left(\left(\frac{\partial f\left(x_i,y_j\right)}{\partial x_i}\right)^2+\left(\frac{\partial f\left(x_i,y_j\right)}{\partial y_j}\right)^2\right)/2}$	The average gradient measures the clarity of the fused image. A larger average gradient indicates more texture details and higher quality of the fused image(Jin and Wang, 2014).
SF	$SF=\sqrt{RF^2+CF^2}$ $RF=\sqrt{\sum_{i=1}^{M}\sum_{j=2}^{N}\left(x_{i,j}-x_{i,j-1}\right)^2}$ $CF=\sqrt{\sum_{i=2}^{M}\sum_{j=1}^{N}\left(x_{i,j}-x_{i-1,j}\right)^2}$	Line spatial frequency reflects image details and texture information. A higher spatial frequency value indicates richer texture details and edge information in the fused image, making it easier for human vision to perceive.
SD	$SD=\sqrt{\frac{1}{MN}\sum_{i=1}^{M}\sum_{j=1}^{N}[H(\mathrm{i},j)-\bar{H}]^2}$ $\bar{H}=\frac{1}{MN}\sum_{i=1}^{M}\sum_{j=1}^{N}H(\mathrm{i},j)$	Standard deviation reflects the richness of fused image information and measures changes in pixel intensity. A higher standard deviation in the fused image corresponds to increased image contrast and richer semantic information.
SCD	$\mathrm{SCD}=r\left(D_1,S_1\right)+r\left(D_2,S_2\right)$ $r\left(D_k,S_k\right)=\frac{\sum_i\sum_j\left(D_k(i,j)-\bar{D}_k\right)\left(S_k(i,j)-\bar{S}_k\right)}{\sqrt{\left(\sum_i\sum_j\left(D_k(i,j)-\bar{D}_k\right)^2\right)\left(\sum_i\sum_j\left(S_k(i,j)-\bar{S}_k\right)^2\right)}}$	Difference correlation involves using the sum of correlations between the input image and the difference image as a quality measure for the fused image. (Aslantas and Bendes, 2015)。
Q<br>abf	$Q^{AB/F}=\frac{\sum_{n=1}^{N}\sum_{m=1}^{M}Q^{AF}(n,m)w_A(n,m)+Q^{BF}(n,m)w_B(n,m)}{\sum_{i=1}^{N}\sum_{j=1}^{M}\left(w_A(i,j)+w_B(i,j)\right)}$ $Q^{XF}(n,m)=Q_g^{XF}(n,m)Q_a^{XF}(n,m)$	Gradient-based fusion performance assesses how well edge information from the original image is preserved in the fused image using local metrics. A smaller value indicates more lost edge information, while a larger value suggests more complete preservation of edge information.
EN	$EN=-\sum_{x=0}^{L}p(x)\log_2 p(x)$	Information entropy reflects the richness of information in an image. Generally, the more information an image contains, the higher its information entropy.

In the AG calculation formula, *f (x, y)* is the image function, and *M* and *N* are the rows and columns of the image, respectively. In the SF calculation formula, x_{ij} represents a point on the fused image. In the SD calculation formula, represents the average grayscale value of the image. In the SCD calculation formula, (D_1 and D_2 represent the difference image, with their respective calculation formulas $D_1=F-S_2$ and $D_2=F-S_1$. This indicates that the difference image between one of the input images (S_2) and the fused image (F) nearly reveals the information conveyed from the other input image (S_1). The function calculates the correlation between S_1 and D_1, S_2 and D_2. Here, k = 1, 2, and represent the average pixel values of S_k and D_k respectively. In the $Q^{AB/F}$ calculation formula, $Q_g^{XF}(n,m)$ and $Q_a^{XF}(n,m)$ represent the edge strength and direction value at the (n,m) position respectively, and w_A and w_B represent the weight. In the EN calculation formula, x represents the grayscale level of the image, and *p(x)* denotes the probability distribution of the grayscale value.
