# Supplementary figures and images for: CFIHL: a variety of chlorophyll a fluorescence transient image datasets of hydroponic lettuce
Source: Front Plant Sci. 2024 Sep 12;15:1414324. doi: 10.3389/fpls.2024.1414324 (PMC11428101; doi:10.3389/fpls.2024.1414324)

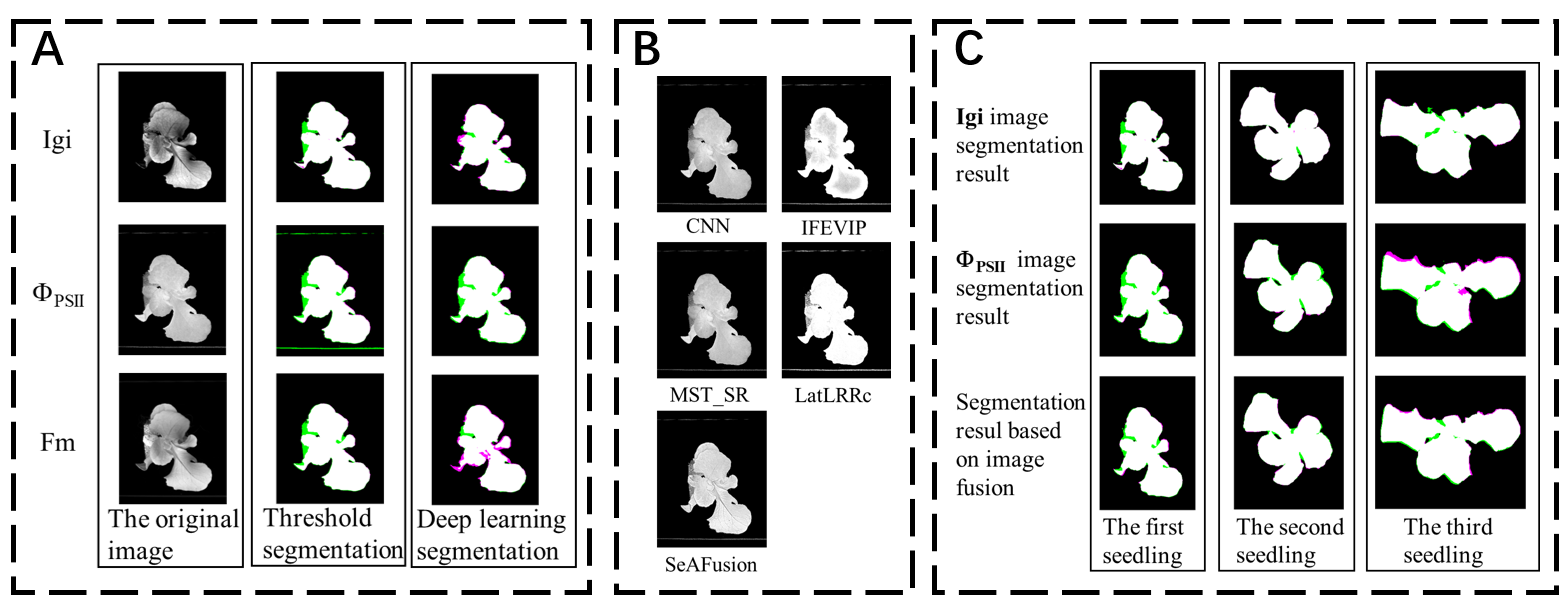

Supplement: Supplementary file 2 [file Image1.png]
